# Comparison of clinical outcomes and complications between endoscopic and minimally invasive transforaminal lumbar interbody fusion for lumbar degenerative diseases: a systematic review and meta-analysis

**DOI:** 10.1186/s13018-024-04549-7

**Published:** 2024-01-27

**Authors:** Abuduwupuer Haibier, Alimujiang Yusufu, Lin Hang, Tuerhongjiang Abudurexiti

**Affiliations:** 1 XinJiang Medical University, Urumqi, 830054 Xinjiang Uygur Autonomous Region People’s Republic of China; 2 Department of Orthopedics of Trauma, The Sixth Affiliated Hospital of Xinjiang Medical University, Orthopedic Hospital of Xinjiang Uygur Autonomous Region, No.39 Wuxing Road, Urumqi, 830002 Xinjiang Uygur Autonomous Region People’s Republic of China

**Keywords:** Degenerative lumbar diseases, Fusion, Endoscopic, Minimally invasive, Transforaminal, Meta-analysis

## Abstract

**Objective:**

This study compares the efficacy and complications of endoscopic transforaminal lumbar fusion (Endo-TLIF) and minimally invasive transforaminal lumbar fusion (MIS-TLIF) in treating lumbar degenerative diseases. It aims to provide reference data for clinical decision-making.

**Methods:**

We identified randomized controlled studies and non-randomized controlled studies on Endo-TLIF and MIS-TLIF for treating lumbar degenerative diseases based on specific inclusion and exclusion criteria. Data were managed with Endnote X9 software and meta-analyzed using Revman 5.3 software. Extracted outcomes included lower back VAS score, lower extremity pain VAS score, low back pain ODI score, complication rate, fusion rate, time to surgery, blood loss, and length of hospital stay.

**Results:**

① Thirteen high-quality studies were included in this meta-analysis, totaling 1015 patients—493 in the Endo-TLIF group and 522 in the MIS-TLIF group. ② Meta-analysis results revealed no significant differences in preoperative, postoperative 6-month, and final follow-up waist VAS scores, lower limb pain VAS score, ODI index, complications, and fusion rate between the two groups (*P* > 0.05). The MIS-TLIF group had a shorter operative time (MD = 29.13, 95% CI 10.86, 47.39, *P* = 0.002) than the Endo-TLIF group. However, the Endo-TLIF group had less blood loss (MD = − 76.75, 95% CI − 111.59, − 41.90, *P* < 0.0001), a shorter hospital stay (MD = − 2.15, 95% CI − 2.95, − 1.34, *P* < 0.00001), and lower lumbar VAS scores both immediately postoperative (≤ 2 week) (MD = − 1.12, 95% CI − 1.53, − 0.71, *P* < 0.00001) compared to the MIS-TLIF group.

**Conclusion:**

Meta-analysis results indicated that Endo-TLIF is similar to MIS-TLIF in terms of long-term clinical outcomes, fusion rates, and complication rates. Although MIS-TLIF has a shorter operation time, Endo-TLIF can significantly reduce blood loss and hospital stay duration. Endo-TLIF offers the advantages of less surgical trauma, reduced blood loss, faster recovery, and early alleviation of postoperative back pain.

## Introduction

Degenerative lumbar diseases are prevalent in middle-aged and elderly patients. Traditional lumbar surgeries often involve significant trauma and extended postoperative recovery periods [1, 2]. Recent advancements in spinal endoscopy have revolutionized this field. Endoscopic transforaminal lumbar interbody fusion, as a novel treatment for lumbar degenerative diseases, is gaining recognition for its benefits, including reduced trauma, shorter recovery time, less postoperative pain, and minimal blood loss [3, 4].

Lumbar interbody fusion is a standard surgical procedure for treating degenerative diseases of the lumbar spine [5]. In 1982, Harms et al. [6] reported that transforaminal lumbar interbody fusion (TLIF) can effectively reduce muscle and nerve root traction injuries.However, concerns persist about TLIF, particularly regarding its limited workspace, restricted surgical field visibility, and high complication rate [7]. Conventional surgical methods, such as posterior approach lumbar interbody fusion and modified transforaminal lumbar interbody fusion, often result in substantial damage to posterior spinal anatomy, significant blood loss, prolonged intraoperative nerve stretching, and extended postoperative bed rest. These factors can lead to complications and adversely impact patient outcomes [8–10]. In contrast, minimally invasive transforaminal lumbar interbody fusion (MIS-TLIF) has gained popularity due to its minimal invasiveness and shorter recovery time [11]. Recently, endoscopic transforaminal lumbar interbody fusion (Endo-TLIF) has emerged as a treatment option for degenerative lumbar diseases. Endo-TLIF offers significant advantages, such as the ability to achieve decompression by removing the intervertebral disk and facilitating endoscopic fusion [12]. In Endo-TLIF, the endoscope is directly inserted into the intervertebral disk space, allowing for complete removal of the cartilage endplate under clear endoscopic vision without damaging the bone endplate. This technique ensures optimal endplate preparation, enhancing interbody fusion [13]. Several studies [14–17] have compared Endo-TLIF and MIS-TLIF, evaluating these procedures from various perspectives to determine the less invasive option.

To the best of our knowledge, comprehensive reports on Endo-TLIF and MIS-TLIF for treating lumbar degenerative diseases are limited both domestically and internationally. Therefore, we conducted a meta-analysis to compare the clinical outcomes and complications of Endo-TLIF and MIS-TLIF in treating these conditions, aiming to provide a theoretical foundation for clinical medical decision-making.

## Material and methods

### Data sources and searches

This systematic review and meta-analysis was conducted in adherence to the Preferred Reporting Items for Systematic Reviews and Meta-Analyzes (PRISMA) guidelines. A comprehensive systematic search was performed across various electronic databases, including PubMed, Cochrane Library, EMbase, CNKI, Wanfang, and VIP database, to identify studies comparing Endo-TLIF and MIS-TLIF in patients with lumbar degenerative diseases. The search was conducted up to May 2023. Keywords used included "degenerative lumbar diseases", "fusion", "endoscopic", "minimally invasive", "transforaminal", and "Meta-analysis", Combined with various operations "AND", "NOT", and "OR". Language restrictions were applied to English and Chinese, and the focus was on human clinical trials.

### Inclusion and exclusion criteria

#### The inclusion criteria were listed as follows

(1) Subjects: Patients diagnosed with degenerative diseases of the lumbar spine; (2) Study Type: Randomized controlled studies (RCTs) or retrospective studies examining the treatment of lumbar degenerative disease with Endo-TLIF and MIS-TLIF; (3) Intervention: The Endo-TLIF group receiving Endo-TLIF as the primary treatment method, with the control group undergoing MIS-TLIF; (4) Outcome Measures: Variables such as lumbar VAS score, lower extremity pain VAS score, ODI score, complication rate, fusion rate, operation time, blood loss, and length of hospital stay; (5) Follow-up Duration: Minimum follow-up period of one year.

#### The following exclusion criteria were used

(1) Involved fewer than 20 cases; (2) Were literature reviews, case reports, or conference abstracts; (3) Lacked complete original data; (4) Were instances of duplicate publication.

### Data extraction

Two independent reviewers extracted data from all included studies using a standardized form to ensure consistency in data collection. Only eligible full-text articles with sufficient data for extraction and pooling were considered. In cases where relevant data were not available in the articles, the authors were contacted via email to request the necessary information. The extracted data included study characteristics such as authors, publication year, study design, sample sizes of different groups, type/classification of fracture, implants used for internal fixation, and follow-up duration. Clinical outcomes included baseline indicators (author, publication time, region, study method, sample size, age, surgical method, and follow-up time) and main outcome indicators (lower back VAS score, lower limb VAS score, lower back ODI score, complication rate, fusion rate, operation time, blood loss, length of hospital stay).

### Outcome measures

(1) Baseline Indicators: These included the author, publication time, region, study method, sample size, age, surgical method, and follow-up time. (2) Main Outcome Indicators: These encompassed lower back VAS score, lower limb VAS score, lower back ODI score, complication rate, fusion rate, operation time, blood loss, and length of hospital stay.

### Data quality assessment

The quality of all included articles was independently assessed by two reviewers. Cohort and case–control studies were evaluated using the Newcastle–Ottawa Scale (NOS). A study scoring ≥ 7 points on the NOS was considered high quality, those scoring 5–6 points were deemed medium quality, and studies scoring < 5 were categorized as low quality. For Randomized Controlled Studies (RCTs), quality assessment was conducted using Review Manager 5.3, focusing on seven key evaluation indexes: random sequence generation, allocation concealment, blinding of participants and personnel, blinding of outcome assessment, completeness of outcome data, selective reporting, and other potential sources of bias. Quality evaluation charts were generated for each study. In cases of disagreement between reviewers, a third researcher was consulted for a final decision.

### Data synthesis and analysis

Meta-analysis was performed using RevMan 5.3 software. For count data, RR was employed as the measure of effect. For continuous data indices, assuming consistency in measurement methods and tools, mean difference (MD) was used as the effect scale, with 95% confidence intervals (CIs) provided for all effect indicators. Heterogeneity among studies was assessed using the *I*^2^ statistic and *P* value: if *P* > 0.1 and I^2^ < 50%, indicating low heterogeneity, a fixed-effect model was used. Conversely, if *P* ≤ 0.1 and *I*^2^ ≥ 50%, indicating high heterogeneity, a random-effects model was employed, supplemented by subgroup and sensitivity Analyzes to identify heterogeneity sources. Sensitivity analysis was performed by sequentially excluding individual studies to assess their impact on overall effect size. Publication bias was evaluated using funnel plots. The threshold for statistical significance was set at *P* ≤ 0.05.

## Results

### Literature search results and literature screening flow

**Fig. 1:**
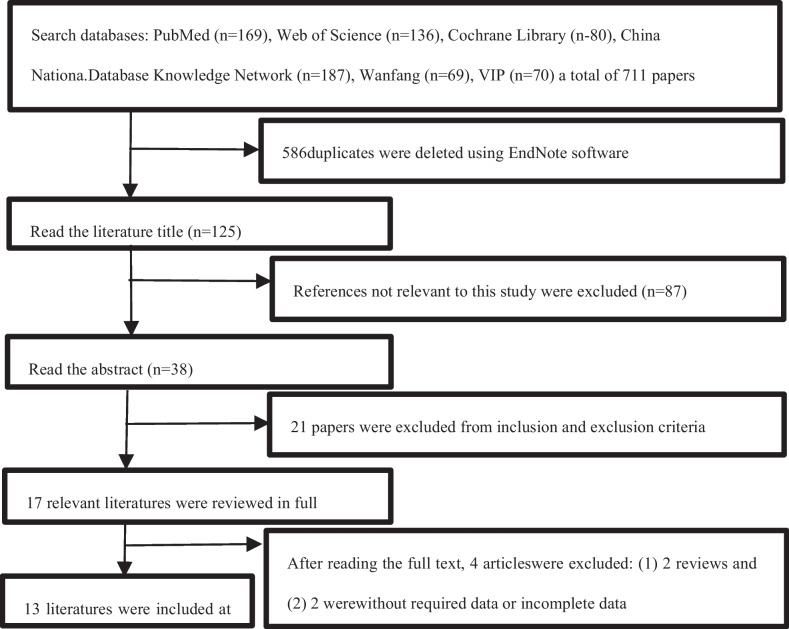
Literature screening process and results

The literature search across various databases yielded a total of 711 articles: 169 from PubMed, 136 from the Web of Science, 80 from Cochrane Library, 187 from the Chinese database Zhihu.com, 69 from Wanfang, and 70 from VIP. After the final screening, 13 literatures [4, 12, 14, 18–27] that met the criteria were selected. Among these, one [22] was a randomized controlled study, and the remaining 12 [4, 12, 14, 18–21, 23–27] were retrospective case–control studies or prospective cohort studies. These studies collectively included a total of 1015 patients with lumbar degenerative disease. The process and results of the literature screening are illustrated in Fig. 1. The basic characteristics of the 13 included studies [4, 12, 14, 18–27] are summarized in Table 1.

**Table 1:** Basic characteristics of the 13 studies were included

No.	Author (year)	Country	Study type	Sex (male/female)		Age		Operation level		Follow-up/month	Outcome measures
				Endo-TLIF	MIS-TLIF	Endo-TLIF	MIS-TLIF	Endo-TLIF	MIS-TLIF		
1	Lim, 2017 [25]	Korea	CC	13/10	22/14	62.7 ± 4.1	58.2 ± 8.2	L2-3**:** 1 L3-4**:** 2 L4-5**:** 12 L5-S1**:** 7	L2-3**:** 2 L3-4**:** 4 L4-5**:** 20 L5-S1**:** 10	≥ 12	①③④⑥
2	AO, 2020 [4]	China	PCS	16/19	22/18	52.80 ± 7.50	53.68 ± 7.24	L3-4**:** 1 L4-5**:** 25 L5-S1**:** 9	L3-4**:** 1 L4-5**:** 19 L5-S1**:** 2	≥ 12	①②③④⑤⑥⑦⑧
3	Gatam, 2020 [26]	Indonesia	CC	26/46	28/45	55.1 ± 5.12	52.3 ± 6.13	L3-4**:** 8 L4-5**:** 56 L5-S1**:** 8	L3-4**:** 10 L4-5**:** 48 L5-S1**:** 5	≥ 12	④
4	Kim, 2020 [19]	Korea	PCS	17/15	25/30	70.5 ± 8.26	67.3 ± 10.7	L2-3**:** 1 L3-4**:** 3 L4-5**:** 20 L5-S1**:** 8	L2-3**:** 0 L3-4**:** 2 L4-5**:** 46 L5-S1**:** 7	18.4 (14–38)	①②③④⑤⑥⑧
5	Zhao, 2021 [12]	China	PCS	23/17	20/18	56.93 ± 1.66	57.01 ± 0.95	L3-4**:** 7 L4-5**:** 24 L5-S1**:** 9	L3-4**:** 8 L4-5**:** 22 L5-S1**:** 8	30.7 (24–34)	④⑤⑥⑦⑧
6	Lv, 2021 [22]	China	RCT	30/24	26/22	54.96 ± 13.16	53.98 ± 11.51	L3-4**:** 9 L4-5**:** 31 L5-S1**:** 14	L3-4**:** 8 L4-5**:** 24 L5-S1**:** 16	≥ 18	①②③④⑤⑥⑦⑧
7	Kang, 2021 [23]	Korea	PCS	17/30	17/15	66.87 ± 10.41	66.38 ± 9.45	L2-3**:** 4 L3-4**:** 7 L4-5**:** 34 L5-S1**:** 20	L2-3**:** 1 L3-4**:** 9 L4-5**:** 22 L5-S1**:** 11	15.01 ± 2.53	④⑥⑦⑧
8	Zhang, 2021 [14]	China	CC	12/20	14/16	53.1 ± 12.8	55.7 ± 14.2	L3-4**:** 3 L4-5**:** 28 L5-S1**:** 1	L3-4**:** 1 L4-5**:** 27 L5-S1**:** 2	≥ 12	①②③④⑤⑥⑦
9	Chang, 2022 [24]	China	PCS	26	32	57.2 ± 13.5	56.1 ± 12.1	L4-5**:** 26	L4-5**:** 32	≥ 18	①②③④⑥⑦
10	Xue, 2022 [21]	China	CC	20	20	46.3 ± 17.2	48.4 ± 13.6	L3-4**:** 6 L4-5**:** 14	L3-4**:** 5 L4-5**:** 15	≥ 18	①②③④⑤⑥⑦⑧
11	Shi, 2023 [20]	China	CC	32	32	59.3 ± 6.2	59.2 ± 5.5	L4-5**:** 32	L4-5**:** 32	≥ 18	①②③④⑥⑦
12	Ge, 2022 [18]	China	CC	21/20	19/24	59.6 ± 7.6	62.7 ± 10.4	L3-4**:** 1 L4-5**:** 33 L5-S1**:** 7	L3-4**:** 2 L4-5**:** 35 L5-S1**:** 6	≥ 18	①②③④⑤⑥⑦
13	Han, 2022 [27]	China	CC	18/21	23/20	60.35 ± 8.04	60.98 ± 6.62	L3-4**:** 9 L4-5**:** 27 L5-S1**:** 3	L3-4**:** 10 L4-5**:** 24 L5-S1**:** 9	≥ 18	①②③④⑤⑥⑦⑧

① lumbar VAS score; (2) lower extremity pain VAS score; ③ ODI score for low back pain; ④ Complications; ⑤ Fusion rate; ⑥ Operation time; ⑦ Blood loss; ⑧ Length of stay, *PCS* prospective cohort study, *RCT* randomized clinical trial, *CC* case–control

### Quality evaluation results of included studies

**Fig. 2:**
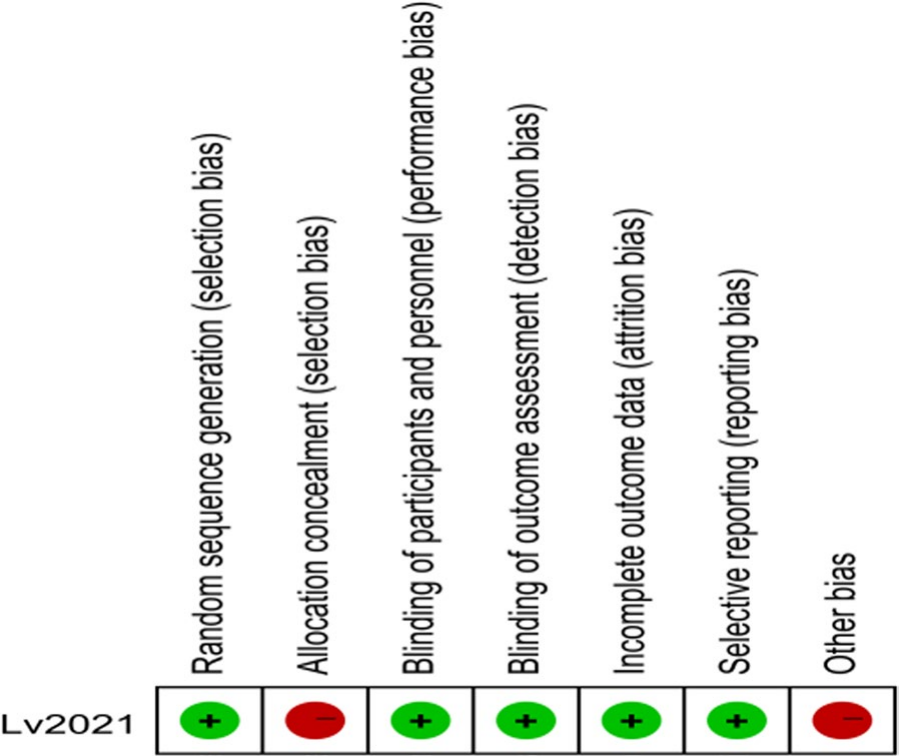
General diagram of quality assessment of the included literature

**Fig. 3:**
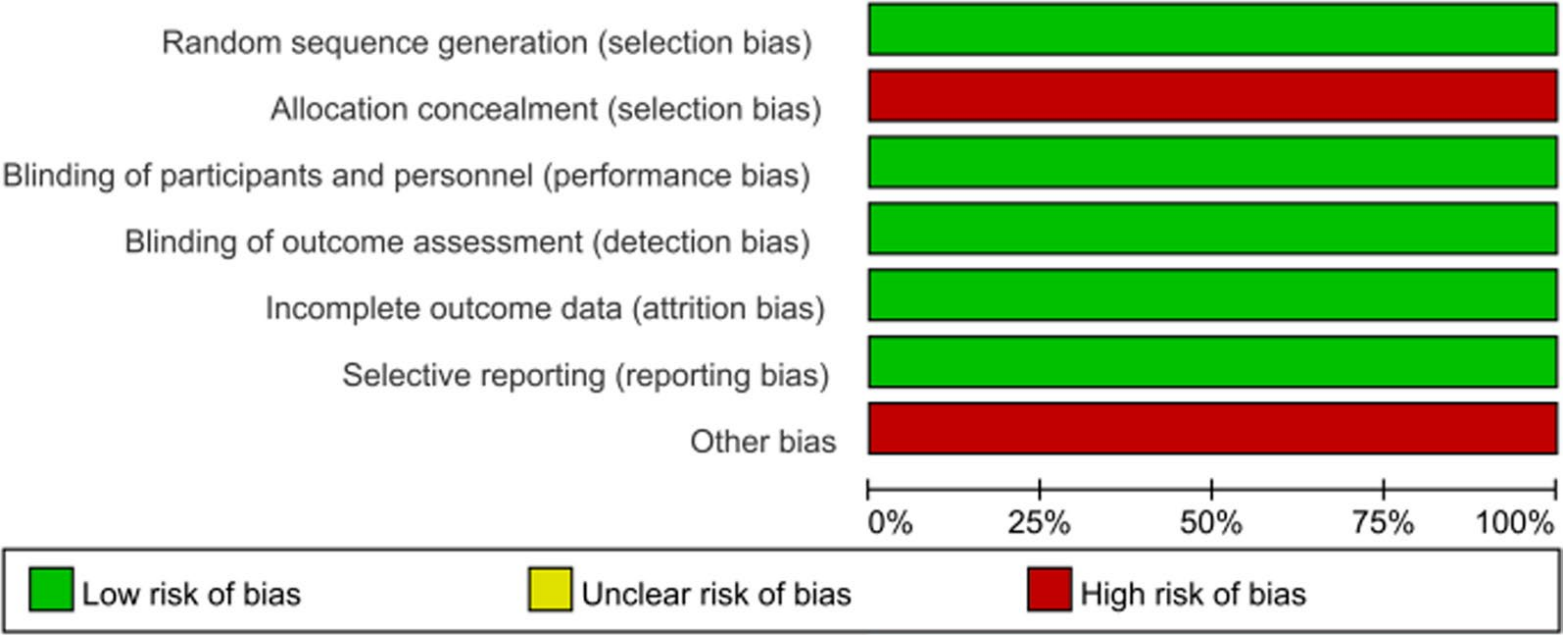
Statistical chart of percentage of quality assessment items for the included literature

The quality of the included studies was assessed using RevMan 5.3 software. Specifically, the last included randomized controlled study [22] was evaluated for bias risk and quality. According to Figs. 2, 3, the Lv, 2021 study [22], was found to have a low risk of bias and was classified as medium–high quality literature. Table 2 indicates that the overall quality of the literature included in the analysis was high. The retrospective case–control studies or prospective cohort studies were assessed using the Newcastle–Ottawa Scale (NOS). Out of the 12 retrospective case–control studies or prospective cohort studies [4, 12, 14, 18–21, 23–27], all were evaluated using the NOS scale and classified as high-quality studies. No low-quality studies were included in the meta-analysis, as detailed in Table 2.

**Table 2:** Literature quality assessment

Author (year)	Selection	Comparability	Exposure	Total score	Quality rating
Lim, 2020 [25]	3	2	3	8	High quality
AO, 2020 [4]	3	2	3	8	High quality
Gatam, 2020 [24]	3	2	2	7	High quality
Kim, 2020 [19]	3	2	3	8	High quality
Zhao, 2021 [12]	3	2	2	7	High quality
Kang, 2021 [23]	3	2	2	7	High quality
Zhang, 2021 [14]	3	2	3	8	High quality
Chang, 2022 [24]	3	2	2	7	High quality
Xue, 2022 [21]	3	2	3	8	High quality
Shi, 2023 [20]	3	2	2	8	High quality
Ge, 2022 [18]	3	3	2	8	High quality
Han, 2022 [27]	3	2	2	7	High quality

### Outcomes of the meta-analysis

#### Differences in lumbar and dorsal VAS scores among groups

**Fig. 4:**
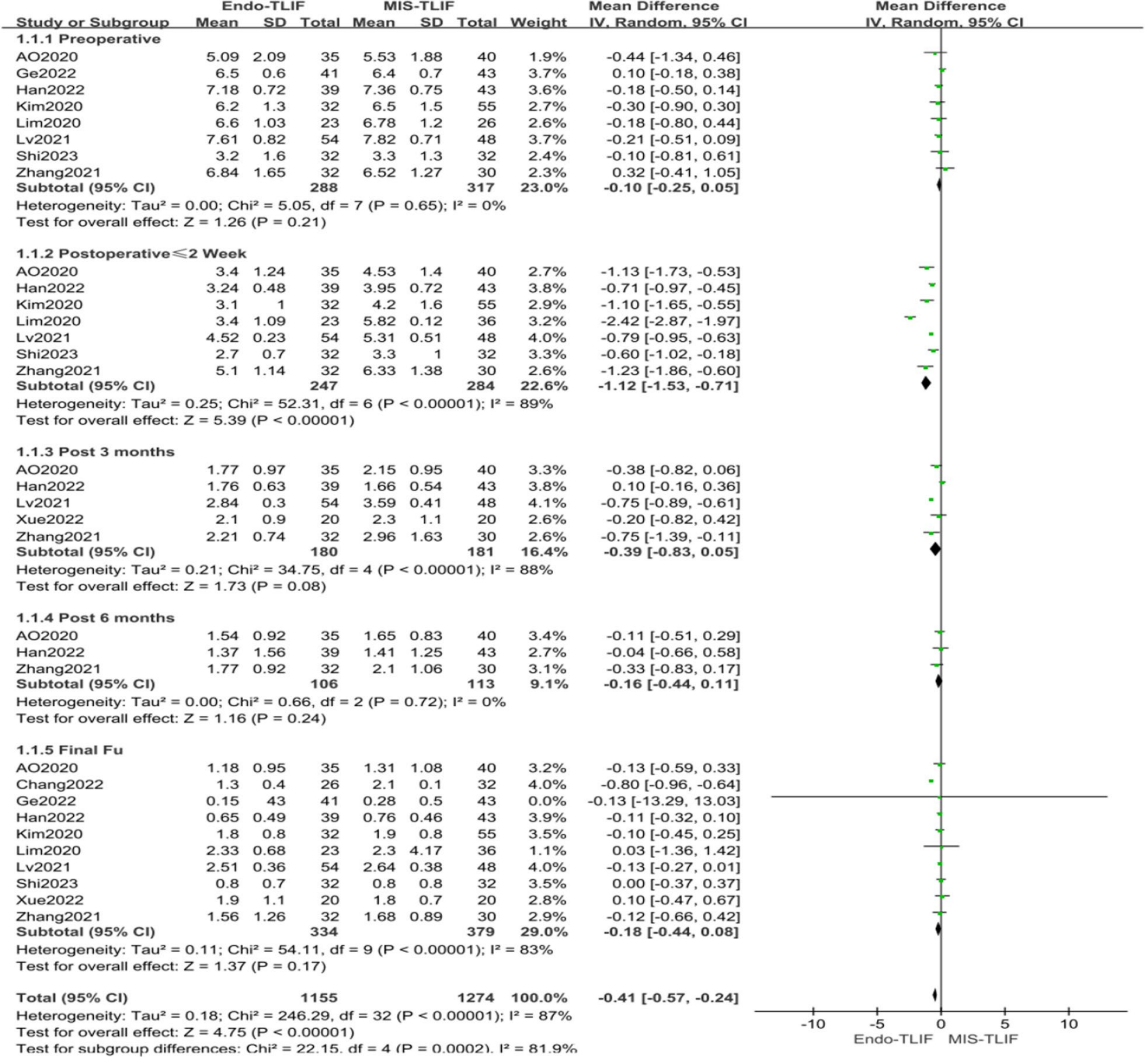
Comparison of VAS scores for back pain between both groups

The lumbar VAS score was reported in ten literatures [4, 14, 18–22, 24, 25, 27]. Preoperative lumbar VAS scores were documented in eight studies [4, 14, 18–20, 22, 23, 27], with a heterogeneity test showing low variation (*P* = 0.65, *I*^2^ = 0%). Seven literatures [4, 14, 19, 20, 22, 25, 27] provided postoperative lumbar and dorsal VAS scores (≤ 2 weeks), revealing high heterogeneity (*P* < 0.00001, *I*^2^ = 89%). Five studies [4, 14, 21, 22, 27] reported lumbar and dorsal VAS scores three months after surgery, indicating moderate heterogeneity (*P* < 0.00001, *I*^2^ = 88%). Three studies [4, 14, 27] provided data on lumbar and dorsal VAS scores six months post-surgery, showing low heterogeneity (*P* = 0.72, *I*^2^ = 0%). Ten studies [4, 14, 18–22, 24, 25, 27] reported on these scores at the last follow-up, with significant heterogeneity observed (*P* < 0.00001, *I*^2^ = 83%). Due to overall high heterogeneity (*P* < 0.00001, *I*^2^ = 87%), a random effects model was employed for the meta-analysis. The meta-analysis results revealed that the Endo-TLIF group's VAS scores ≤ 2 weeks were significantly lower than those of the MIS-TLIF group, with mean differences (MD) of − 1.12 [95% CI − 1.53, − 0.71, *P* < 0.00001]. However, there were no significant differences in the lumbar and dorsal VAS scores between the groups preoperatively, at 3 months postoperatively, at 6 months postoperatively, and at the last follow-up, with MDs of − 0.10 [95% CI − 0.25, 0.05, *P* = 0.21], − 0.39 [95% CI − 0.83, 0.05, *P* = 0.08], − 0.16 [95% CI − 0.44, 0.11, *P* = 0.24],and − 0.18 [95% CI − 0.44, 0.08, *P* = 0.17], respectively. Overall, the lumbar VAS scores of the Endo-TLIF group were significantly lower than those of the MIS-TLIF group [MD = − 0.41, 95% CI − 0.57, − 0.24, *P* < 0.00001], as shown in Fig. 4. However, the included studies exhibited heterogeneity, necessitating an analysis of the sources of this heterogeneity, as detailed in Table 3.

#### Differences in lower extremity pain VAS scores among groups

**Fig. 5:**
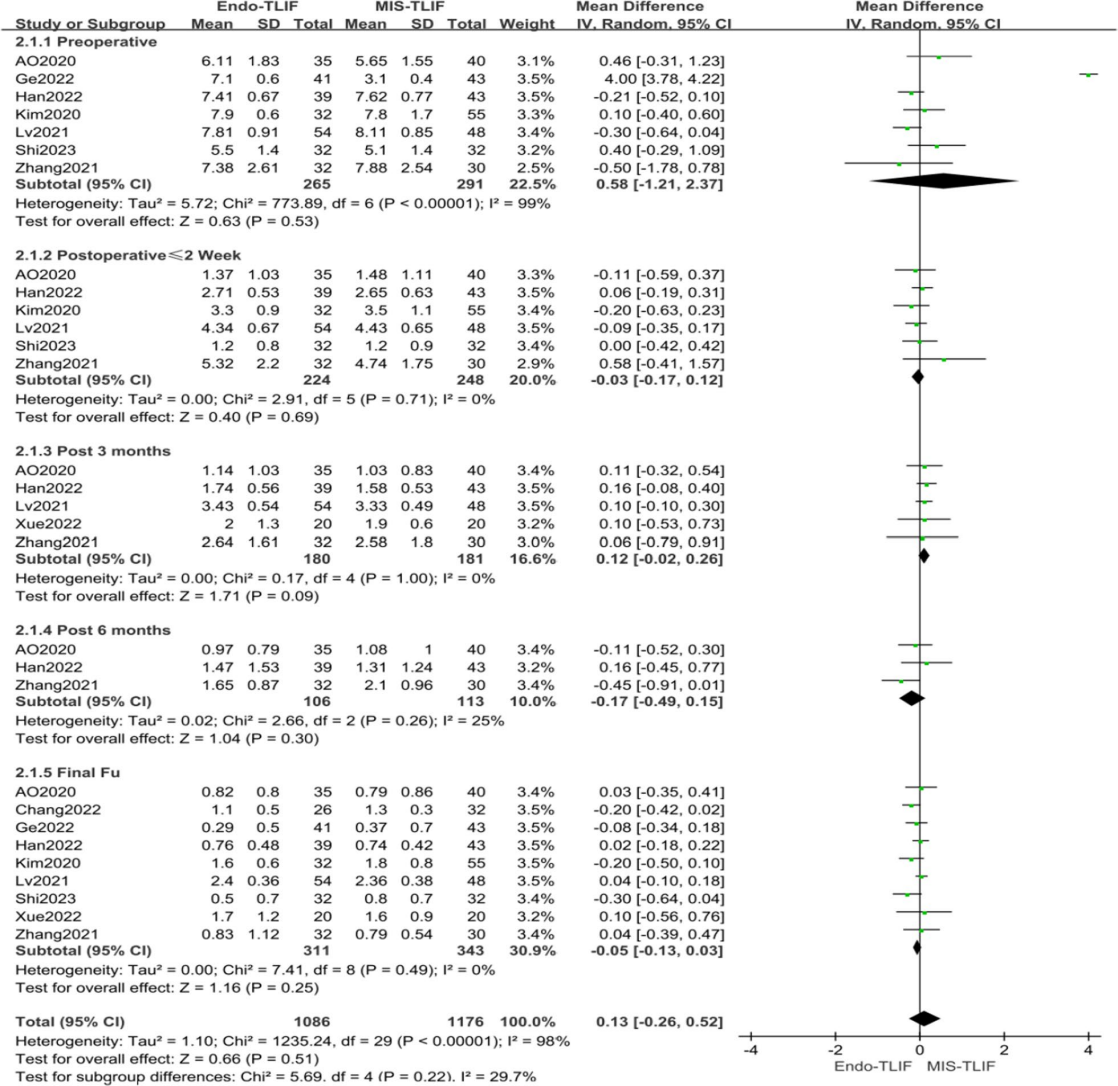
Comparison of VAS scores for lower limb pain between both groups

Nine literatures [4, 14, 18–22, 24, 27] reported on the VAS score for lower extremity pain. Seven of these [4, 14, 18–20, 22, 27] included pre-surgical VAS scores, demonstrating significant heterogeneity (*P* < 0.00001, *I*^2^ = 99%). Six studies [4, 14, 19, 20, 22, 27] reported lower extremity pain VAS scores post-surgery (≤ 2 weeks), with a heterogeneity test indicating low variation (*P* = 0.71, *I*^2^ = 0%). Five literatures [4, 14, 21, 22, 27] provided VAS scores for lower extremity pain three months after surgery, showing no heterogeneity (*P* = 1.00, *I*^2^ = 0%). Three articles [4, 14, 27] reported on these scores six months post-surgery, with some heterogeneity (*P* = 0.26, *I*^2^ = 25%). Lastly, nine articles [4, 14, 18–22, 24, 27] reported on the scores at the last postoperative follow-up, indicating low heterogeneity (*P* = 0.49, *I*^2^ = 0%). Consequently, a fixed effect model was applied for the meta-analysis. The meta-analysis results revealed no significant difference in the VAS scores for lower extremity pain between the MIS-TLIF and Endo-TLIF groups at preoperative, postoperative (≤ 2 weeks), 3 months, 6 months, and last follow-up stages. The MDs were 0.58 [95% CI − 1.21, 2.37, *P* = 0.53], − 0.03 [95% CI − 0.17, 0.12, *P* = 0.69], 0.12 [95% CI − 0.02, 0.26, *P* = 0.09], − 0.17 [95% CI − 0.49, 0.15, *P* = 0.30], and − 0.05 [95% CI − 0.13, 0.03, *P* = 0.25] respectively, as shown in Fig. 5. Overall, there was no significant difference in lower extremity pain VAS scores between the MIS-TLIF and Endo-TLIF groups [MD = 0.13, 95% CI − 0.26, 0.52, *P* = 0.51], as depicted in Fig. 8. However, the studies included showed heterogeneity, necessitating further analysis of the sources of this heterogeneity, as outlined in Table 3.

**Table 3:** The sensitivity analysis excluded the studies considered to contribute the most heterogeneity

Results and subgroup analysis	Number of studies	Exclusion study	Heterogeneity			*P* (overall effect test)
			*x*^2^	*I*^2^, %	*P*	
1.1 Lumbar VAS score						
1.1.1Postoperative ≤ 2 weeks	7	Lim, 2022	5.43	8%	0.37	< 0.00001
1.1.2 Post 3 months	5	Han, 2022	5.05	41%	0.17	< 0.00001
1.1.3 Final FU	10	Chang, 2022	0.98	0%	1.00	= 0.04
1.2 Preoperative (lower extremity pain VAS score)	7	Ge, 2022	0.02	27%	0.23	= 0.58
1.3 ODI score						
1.3.1 Post 3 months	5	Zhang, 2021	3.44	13%	0.33	= 0.17
1.3.1 Post 6 months	3	Zhang, 2021	0.00	0%	0.96	= 0.17

#### Differences in the ODI index of each group

**Fig. 6:**
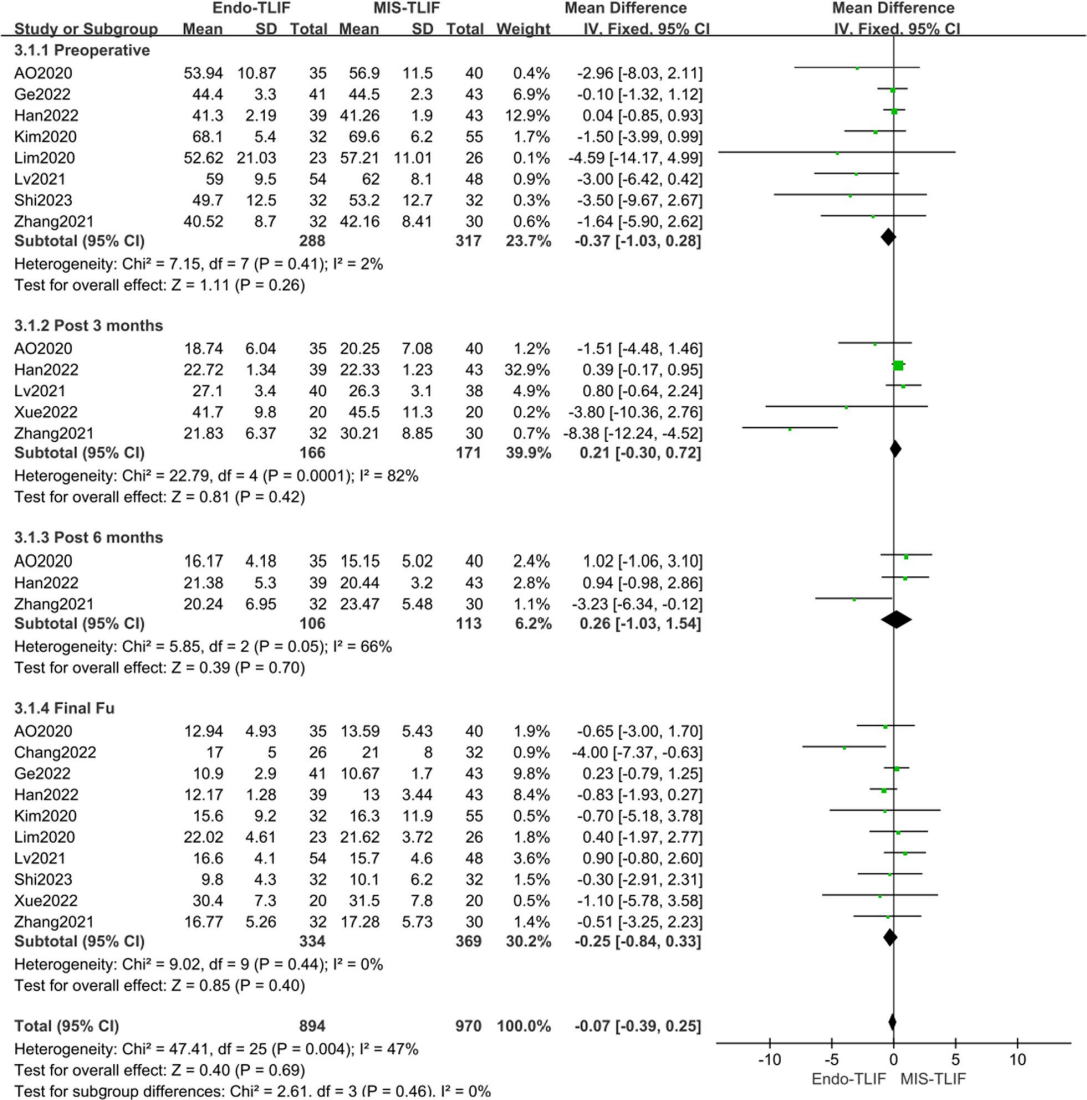
Comparison of the LP ODI scores between the two groups

Ten literatures [4, 14, 18–22, 24, 25, 27] reported on the ODI scores for low back pain. Eight of these [4, 14, 18–20, 22, 25, 27] included ODI scores before surgery, showing no heterogeneity (*P* = 0.41, *I*^2^ = 2%). Five studies [4, 14, 21, 22, 27] reported ODI scores three months post-surgery, with high heterogeneity (*P* = 0.0001, *I*^2^ = 82%). Three literatures [4, 14, 27] provided data on ODI scores six months after surgery, again showing high heterogeneity (*P* = 0.05, *I*^2^ = 66%). Ten studies [4, 14, 18–22, 24, 25, 27] reported ODI scores at the last follow-up, indicating low heterogeneity (*P* = 0.44, *I*^2^ = 0%). Consequently, a fixed-effect model was used for the meta-analysis of ODI scores for low back pain. The meta-analysis results revealed no significant differences in ODI scores between the MIS-TLIF and Endo-TLIF groups preoperatively, at 3 months postoperatively, at 6 months postoperatively, and at the last follow-up. The MDs were − 0.37 [95% CI − 1.03, 0.28, *P* = 0.26], 0.21 [95% CI − 0.30, 0.72, *P* = 0.42], 0.26 [95% CI − 1.03, 1.54, *P* = 0.70], and − 0.25 [95% CI − 0.84, 0.33, *P* = 0.40], respectively. Overall, there was no significant difference in ODI scores for low back pain between the groups [MD = − 0.07, 95% CI − 0.39, 0.25, *P* = 0.69], as depicted in Fig. 6. However, heterogeneity existed in the included studies, necessitating further analysis of the sources of this heterogeneity, as outlined in Table 3.

#### Differences in complication rates among all groups

**Fig. 7:**
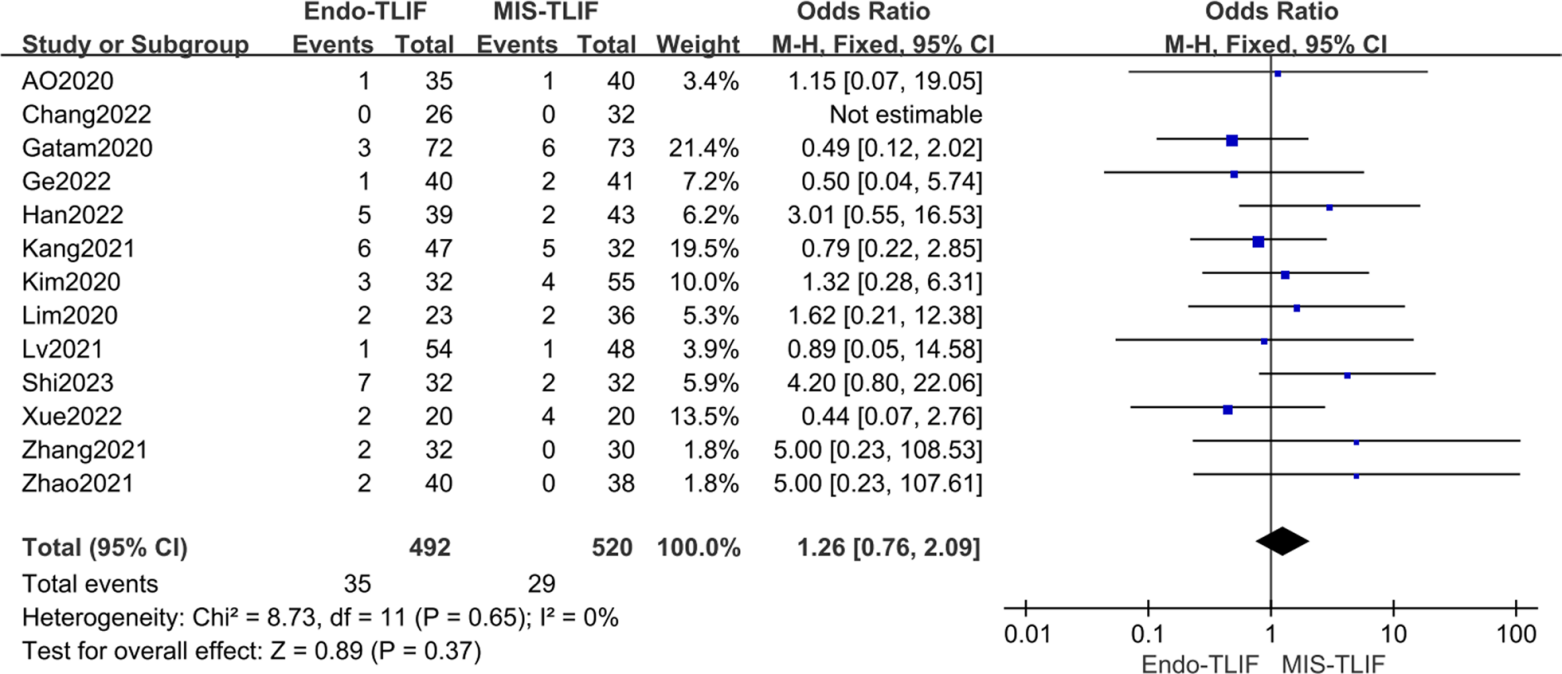
Comparison of the complication rates between the two groups

The meta-analysis included thirteen studies [4, 12, 14, 18–27], revealing no statistical heterogeneity among them (*P* = 0.65, *I*^2^ = 0%). Using a fixed-effect model, no statistically significant difference was found in the incidence of postoperative complications among all groups [OR = 1.26, 95% CI (0.76, 2.09), *P* = 0.37], as shown in Fig. 7 and Table 4.

**Table 4:** Complication rate

Study title	Endo-TLIF		MIS-TLIF	
	Complication	Sample size	Complication	Sample size
Lim, 2017 [25]	2	23	2	36
AO, 2020 [4]	1	35	1	40
Gatam, 2020 [26]	3	72	6	73
Kim, 2020 [19]	3	32	4	55
Zhao, 2021 [12]	2	40	0	38
Lv, 2021 [22]	1	54	1	48
Kang, 2021 [23]	6	47	5	32
Zhang, 2021 [14]	2	32	0	30
Chang, 2022 [24]	0	26	0	32
Xue, 2022 [21]	2	20	4	20
Shi, 2023 [20]	7	32	2	32
Ge, 2022 [18]	1	40	2	41
Han, 2022 [27]	5	39	2	43
Sum	35	492	29	520

#### Differences in fusion rates among groups

**Fig. 8:**
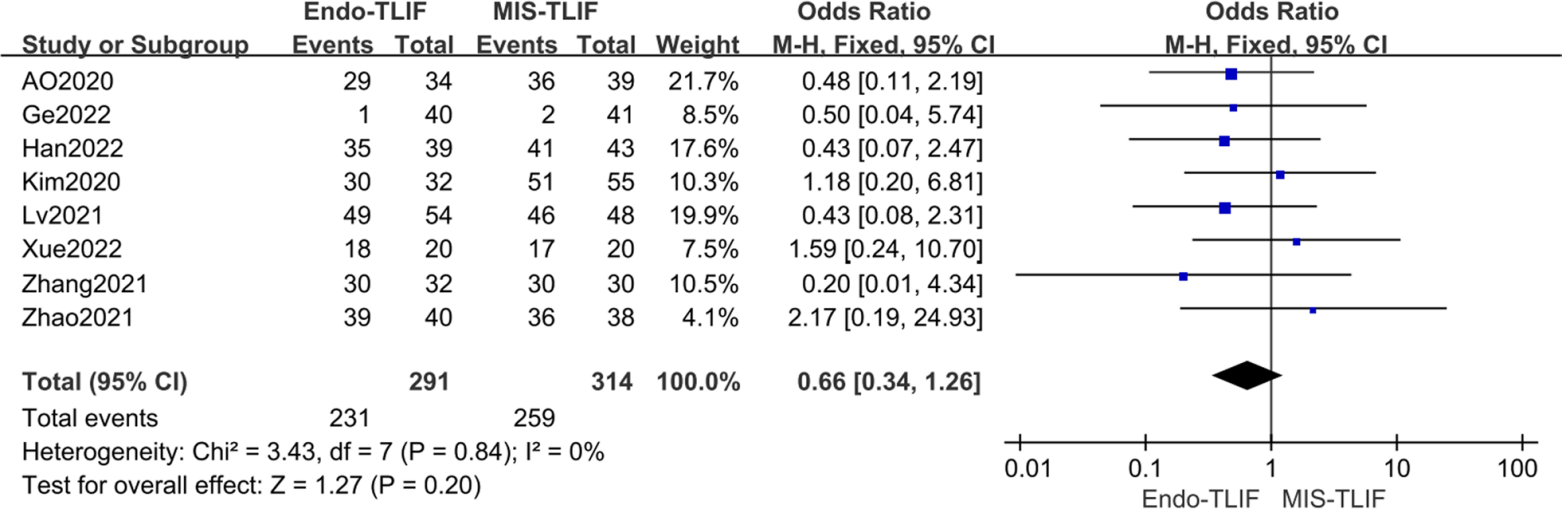
Comparison of the fusion rates between the two groups

Eight studies [4, 12, 14, 18, 19, 21, 22, 27] were included in the analysis of fusion rates, with no statistical heterogeneity observed (*P* = 0.84, *I*^2^ = 0%). Using a fixed-effect model, there was no statistically significant difference in the postoperative fusion rate among all groups [OR = 0.66, 95% CI (0.34, 1.26), *P* = 0.20], as illustrated in Fig. 8.

#### Differences in operation time among groups

**Fig. 9:**
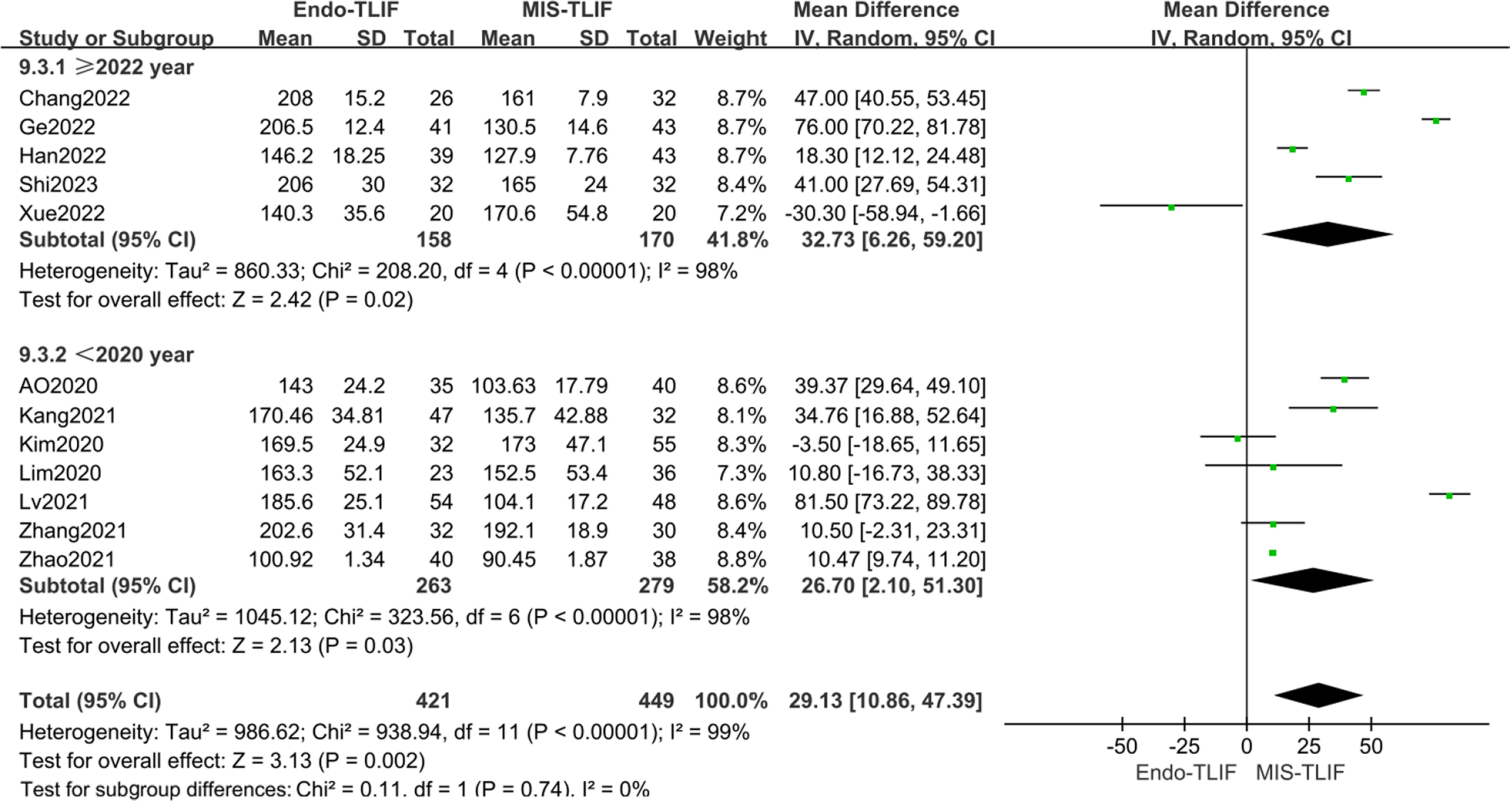
Comparison of the operative time between the two groups

Twelve studies [4, 12, 14, 18–25, 27] reported on operation time. The meta-analysis revealed significant statistical heterogeneity among these studies (*P* < 0.00001, *I*^2^ = 99%). Sensitivity analysis identified the publication year of the studies as a potential source of heterogeneity. Subgroup analysis was conducted based on the publication year of the literature. The ≥ 2022 group [18, 20, 21, 24, 27] showed no heterogeneity (*P* < 0.00001, *I*^2^ = 98%), whereas the < 2022 group [4, 12, 14, 19, 22, 23, 25] exhibited high heterogeneity (*P* < 0.00001, *I*^2^ = 98%). The conclusion remained consistent with the overall pooled results. The combined subgroup analysis indicated that there was a statistically significant difference in operation time between the two groups. Specifically, the operation time in the Endo-TLIF group was significantly higher than that in the MIS-TLIF group, with MDs of 32.73 [95% CI 6.26, 59.20, *P* = 0.002] for the ≥ 2022 group and 26.70 [95% CI 2.10, 51.30, *P* = 0.03] for the < 2022 group, as shown in Fig. 9. Although subgroup analysis was done, no significant factors of heterogeneity were found.

#### Differences in blood loss among groups

**Fig. 10:**
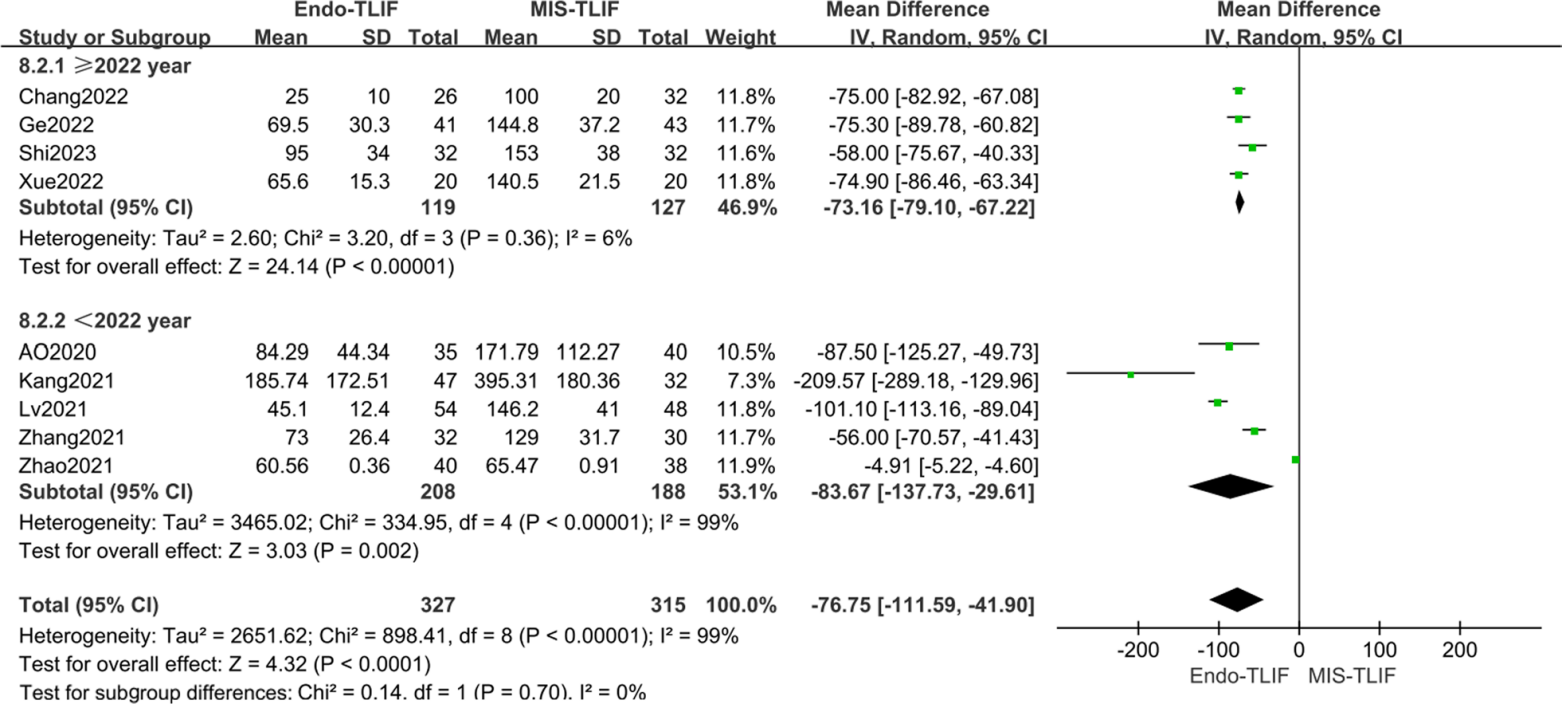
Comparison of blood loss in the two groups

Nine articles [4, 12, 14, 18, 20–24] reported on blood loss. Meta-analysis showed significant statistical heterogeneity among these studies (*P* < 0.00001, *I*^2^ = 99%). Sensitivity analysis identified the publication year of the studies as a potential source of heterogeneity. Subgroup analysis was conducted based on the publication year of the literature. The 2022 group [18, 20, 21, 24] showed no heterogeneity (*P* = 0.36, *I*^2^ = 6%), whereas the < 2022 group [4, 14, 19, 22, 23] exhibited high heterogeneity (*P* < 0.00001, *I*^2^ = 99%). The conclusion remained consistent with the overall pooled results. The combined subgroup analysis indicated that there was a statistically significant difference in blood loss between the two groups. Specifically, the blood loss in the Endo-TLIF group was significantly lower than that in the MIS-TLIF group, with MDs of − 73.16 [95% CI − 79.10, − 67.22, *P* < 0.00001] for the 2022 group and − 83.67 [95% CI − 137.73, − 29.61, *P* = 0.002] for the < 2022 group, as shown in Fig. 10.

#### Differences in hospital stay among groups

**Fig. 11:**
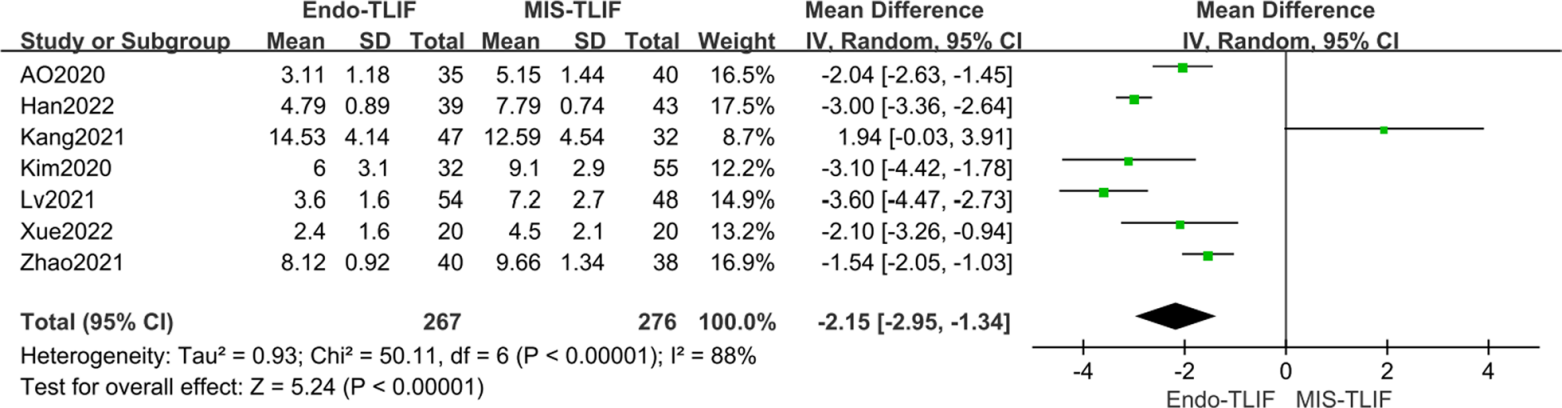
Comparison of the length of hospital stay between the two groups

In the analysis of hospital stay lengths, six studies [4, 12, 19, 21–23, 27] were included. The meta-analysis revealed significant statistical heterogeneity among these studies (*P* < 0.00001, *I*^2^ = 85%). A random effects model was used due to this high heterogeneity. The results indicated a significant difference in hospital stay lengths between the two groups [MD = − 1.95, 95% CI (− 2.90, − 1.00), *P* < 0.0001], with the Endo-TLIF group having a significantly shorter stay compared to the MIS-TLIF group. The persistence of high heterogeneity even after excluding each study individually suggests that these results are reliable. The sources of heterogeneity may include differences in surgeon expertise, the severity of lumbar degeneration diseases, and patient health conditions. Detailed findings are illustrated in Fig. 11.

### Sensitivity analysis

To verify the reliability of the results in this study, sensitivity Analyzes were conducted on various outcome indicators, including the lumbar VAS score (≤ 2 weeks after surgery, 3 months after surgery, and at the last follow-up time), preoperative VAS score of lower limb pain, and the ODI score for low back pain 3 months post-surgery. The Analyzes aimed to identify the sources of heterogeneity, particularly focusing on the length of follow-up time. For the preoperative lumbar VAS score, the effect size changed directionally after the exclusion of Lim, 2017 et al. [25] (*I*^2^ = 8%, *P* = 0.37). No other literature had a significant impact on the overall effect size, suggesting that Lim, 2017 et al. [25] might be the source of heterogeneity. Regarding the lumbar VAS score 3 months post-surgery, the effect size changed directionally (*I*^2^ = 41%, *P* = 0.17) after removing Lv, 2021 et al. [22]. Similarly, for the lumbar VAS score at the last follow-up, the effect size changed directionally after excluding Chang, 2022 et al. [24] (*I*^2^ = 0%, *P* = 1.00). Details of these findings are provided in Table 3. The heterogeneity of the preoperative lower extremity pain VAS score was primarily derived from Ge et al. [18]. Removing this study decreased the heterogeneity among the remaining studies (*I*^2^ = 27%, *P* = 0.58). For the ODI score 3 months post-surgery, the primary source of heterogeneity was identified as the study by Zhang et al. [14]. Excluding this literature resulted in a decrease in heterogeneity and a directional change in effect size (*I*^2^ = 13%, *P* = 0.33).Similarly, for the ODI score 6 months post-surgery, the effect size changed directionally after excluding Zhang et al. [14] (*I*^2^ = 0%, *P* = 0.96). Additionally, the results indicated high heterogeneity in operation time and hospital stay. Even after excluding each study one by one, the heterogeneity remained relatively unchanged, affirming the reliability of the findings. The sources of this heterogeneity could be attributed to variations in surgeon expertise, the nature of lumbar degenerative diseases, and differences in patient health.

### Publication bias analysis

**Fig. 12:**
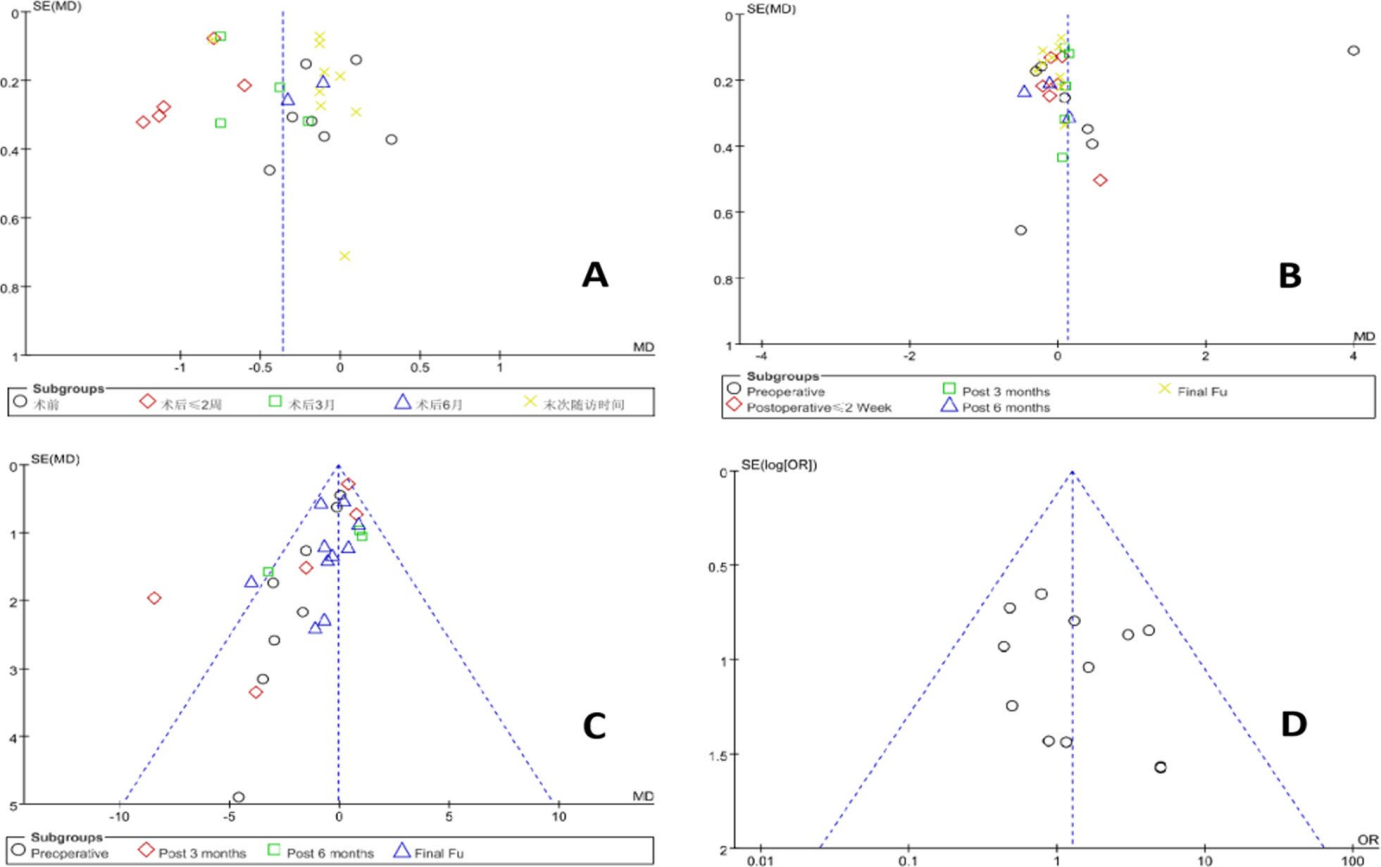
Funnel plots of outcome indicators. **A** Lumbar VAS score; **B** lower extremity pain VAS score; **C** ODI score for low back pain; **D** complications

The examination of publication bias in this study involved the use of RevMan 5.3 software to generate funnel plots for key observational indicators: lumbar VAS score, VAS score for lower extremity pain, ODI score for lumbar pain, and complication rate. The symmetry observed in the funnel plot for the lumbar VAS score suggests a lower likelihood of publication bias. However, asymmetries and gaps in the funnel plots for the ODI score and complication rate indicators for low back pain indicate the possibility of publication bias in these areas. Specifically, the lower limb pain VAS score showed asymmetry and a gap in the lower left corner, along with one study lying outside the 95% confidence interval, suggesting potential bias. This aspect of the study highlights the need for cautious interpretation of these results and points to the necessity of a broader literature search in future studies.Detailed findings are illustrated in Fig. 12**.**

## Discussion

### Summary of evidence

The evolution of lumbar surgery techniques has been significant, especially with the advent of minimally invasive approaches like transforaminal lumbar fusion, minimally invasive transforaminal lumbar fusion, and oblique lateral lumbar fusion. These techniques offer advantages over traditional open surgery, including reduced trauma, shorter recovery times, less postoperative pain, and decreased blood loss. The continuous advancements in endoscopic equipment and techniques have expanded the indications for full endoscopic lumbar surgery, introducing methods like endoscopic spinal canal decompression. These innovations can yield results comparable to traditional surgery while minimizing soft tissue injury, thus facilitating faster postoperative recovery [28–31]

Since 2010, percutaneous endoscopic transforaminal lumbar fusion (Endo-TLIF) has gained popularity for treating lumbar degenerative diseases such as lumbar disk herniation, lumbar spinal stenosis, and lumbar spondylolisthesis. This technique allows for complete endoscopic diskectomy, spinal canal and foraminal decompression, and interbody fusion through endoscopic and working channels [19, 31, 32]. However, Endo-TLIF is not without limitations. It often requires repeated fluoroscopy during surgery, exposing patients and doctors to higher doses of radiation. Furthermore, due to the complex anatomy of the foramina, the procedure demands surgeons with extensive experience in endoscopic decompression surgery [33].

This paper focused on comparing Endo-TLIF and MIS-TLIF in treating lumbar degenerative diseases, evaluating various parameters such as lumbar VAS score, lower extremity pain VAS score, low back pain ODI score, complication rate, fusion rate, operation time, blood loss, and length of hospital stay. A total of 12 studies were included, most of which were assessed as high-quality literature. The meta-analysis indicated that Endo-TLIF and MIS-TLIF are similar in terms of long-term clinical outcomes, fusion rates, and complication rates. Although the MIS-TLIF group showed shorter operation times, the Endo-TLIF group had significant advantages in reducing blood loss and shortening hospital stays.

#### Clinical efficacy

The meta-analysis provided insightful findings on the clinical efficacy of Endo-TLIF compared to MIS-TLIF, particularly in terms of pain reduction. It was observed that the lumbar VAS (Visual analog Scale) score was significantly lower in the Endo-TLIF group than in the MIS-TLIF group, especially within the first 2 weeks and 3 months following surgery. However, no significant differences were found in VAS scores for lower limb pain and ODI scores for low back pain. This pattern was consistent at both day 1 and 3 months post-surgery, where lower back VAS scores were notably lower in the Endo-TLIF group. Both MIS-TLIF and Endo-TLIF groups showed a significant reduction in VAS and ODI scores from preoperative levels, and this reduction was consistent over the observation period. According to Zhu et al. [34], the VAS for the Endo-TLIF group was significantly lower than that for the MIS-TLIF group within 2 weeks and 2–3 months post-surgery. However, no significant differences were observed in the VAS and ODI scores at other follow-up points, including over a 2-year follow-up, indicating similar long-term outcomes in terms of pain and functional improvement.

#### Complications

Compared with MIS-TLIF, Endo-TLIF is characterized by its minimally invasive nature, utilizing 4–5 small incisions of about 1cm each. This approach allows for a gradual expansion of the muscles to access the operation area, with minimal removal of the articular process bone and no use of electric knives. This method results in less muscle and soft tissue damage, promoting quicker postoperative recovery and enabling patients to walk as early as the first day after surgery. It also leads to a reduced incidence of complications such as hypostatic pneumonia, deep vein thrombosis, and pulmonary embolism, along with a shorter postoperative hospital stay [35–38].

Despite these advantages, Shi et al. [20] reported a higher complication rate in the Endo-TLIF group compared to the MIS-TLIF group, challenging the notion that endoscopic fusion surgery is straightforward. This complexity underscores the need for surgeons to have extensive experience in both open lumbar fusion surgery and endoscopic non-fusion surgery before undertaking Endo-TLIF procedures. Additionally, a recent study [39] indicated a higher occurrence of paresthesia in Endo-TLIF compared to conventional endoscopic surgery.

However, this study found no significant difference in the overall incidence of complications between the Endo-TLIF and MIS-TLIF groups. While the rates of complications may be comparable, Endo-TLIF has the advantage of causing less blood loss, inflicting less injury to normal soft tissues, requiring a shorter hospital stay, and allowing for a faster recovery. It's important to note that both Endo-TLIF and MIS-TLIF can lead to complications like dural sac tears, nerve root sleeve tears, intramuscular hematomas, intervertebral space infections, and severe epidural adhesions. Nevertheless, our findings suggest that Endo-TLIF does not increase the risk of these complications compared to MIS-TLIF and may even prevent issues like severe epidural adhesions.

In summary, while postoperative complications do not significantly limit the choice of operation between Endo-TLIF and MIS-TLIF, a comprehensive understanding of their safety profiles is crucial. Further studies and detailed Analyzes of different types of complication events are needed.

#### Fusion rate

Regarding the fusion rates, studies [2, 40] have shown that MIS-TLIF achieved high fusion rates, with 88.9% at 1 year and 96.0% at 3 years post-surgery. Data from Heo et al. [37] indicated that the Endo-TLIF group had a fusion rate of 73.9% one year after surgery. These findings suggest that the fusion rates are similar between the two groups [41]. Zhu et al. [34] reported that the fusion rate was 89% in the Endo-TLIF group and 91% in the MIS-TLIF group at the last follow-up, showing no statistical significance. In this meta-analysis, which included 7 studies with 523 cases, there were no statistically significant differences in fusion rates between Endo-TLIF and MIS-TLIF (OR = 0.71, 95% CI 0.35, 1.42, *P* = 0.33, *I*^2^ = 0%). These results align with the findings of the current study.

#### Operation time, blood loss and length of stay

This study demonstrated that the operation time for the Endo-TLIF group was longer than for the MIS-TLIF group (*P* = 0.002). Endo-TLIF, being a new technology with a steep learning curve, necessitates prospective guidance to ensure safety and effectiveness. Operators should have substantial experience in percutaneous endoscopic surgery before employing this method. Data from Zhao et al. [12] revealed that, compared to the MIS-TLIF group, the Endo-TLIF group experienced less blood loss, reduced normal tissue damage, shorter hospital stays, and quicker recovery. This approach minimizes pathway trauma by utilizing muscle expansion rather than muscle contraction. Zhu et al.'s [34] meta-analysis, which included 1,475 patients undergoing surgery for lumbar degenerative diseases from 28 studies (549 Endo-TLIF cases and 927 MIS-TLIF cases), indicated that Endo-TLIF significantly reduced intraoperative blood loss compared to MIS-TLIF (mean intraoperative blood loss was 101.1 ml and 174 ml, respectively, and average blood loss per fusion segment was 92.9 ml and 160.5 ml, respectively). The incidence of complications between the groups was not significantly different (intraoperative complications were 2.2% and 2.5%, and postoperative complications were 7.8% and 10.2%, respectively). Lv et al. [22] reported that although MIS-TLIF had a significantly shorter operation time than Endo-TLIF (mean operation times were 146.1 min and 104.1 min, respectively), the Endo-TLIF group significantly reduced blood loss (mean blood loss: 45.1 ml in Endo-TLIF and 146.2 ml in MIS-TLIF) and hospital stay duration (average stays: 3.6 days in Endo-TLIF and 7.2 days in MIS-TLIF), aligning with this study's results. Ao et al.'s [4] prospective cohort study of 75 patients with single-level lumbar degenerative diseases (including single-level lumbar spinal stenosis, Meyerding grade I spondylolisthesis, and lumbar disk herniation) with 35 undergoing Endo-TLIF and 40 MIS-TLIF, found the Endo-TLIF group had less intraoperative blood loss. Kou et al.'s [17] meta-analysis of four studies (273 cases) reported the hospital stay length in the Endo-TLIF group was significantly shorter than in the MIS-TLIF group (WMD = 3.55, 95% CI − 5.54, 1.16, *P* = 0.004, *I*^2^ = 95.7%). These findings are in line with those of the current study.

### Analysis of article results

This study comprehensively analyzed 12 studies involving a total of 933 patients, with 454 patients in the Endo-TLIF group and 479 in the MIS-TLIF group. The meta-analysis and subgroup analysis focused on evaluating various parameters, including lumbar VAS score, lower extremity pain VAS score, low back pain ODI score, complication rate, fusion rate, operative time, blood loss, and length of hospital stay. The findings revealed that in terms of long-term clinical outcomes, fusion rate, and complication rate, Endo-TLIF and MIS-TLIF were similar. However, it was noted that although the operation time for MIS-TLIF was shorter, Endo-TLIF was more effective in reducing blood loss and shortening the duration of hospital stays. The subgroup analysis highlighted that the lumbar VAS score was significantly lower in the Endo-TLIF group compared to the MIS-TLIF group, but there was no significant difference in the VAS score for lower limb pain and the ODI score for low back pain.

### Differences and innovation between this paper and the previous studies

Earlier studies often included fewer than 10 studies, which limited the scope for comprehensive Analyzes such as meta-regression and publication bias. Secondly, unlike previous studies, this research conducted subgroup Analyzes considering complication, fusion rate, and blood loss. These additional layers of analysis may account for differences in the final results, particularly in lower limb VAS score, waist ODI score, and blood loss. Furthermore, this study went beyond the typical scope of analysis by conducting a detailed examination of each index, which also enhances its clinical relevance and instructiveness.

### Limitations of the article

(1) Some of the included literature presented counting data graphically, necessitating manual extraction of mean and standard deviation. This process could introduce errors and potentially affect the authenticity of the results. (2) The majority of the included studies were retrospective and had small sample sizes. Additionally, the follow-up duration in some studies was not long enough, possibly leading to the under-detection of postoperative complications, insufficient statistical power, and reporting bias. (3) The study's evaluation parameters were limited and did not encompass aspects like treatment cost and specific adverse reactions, which are important in a comprehensive assessment of surgical procedures. (4) The follow-up periods in the included studies varied, which might have influenced the comparability of the results. Furthermore, the ongoing controversy over the choice of surgical method underscores the need for more extensive, multi-center, prospective studies to provide clearer guidance.

### Implications for future research

The findings of this meta-analysis suggest that Endo-TLIF and MIS-TLIF have comparable clinical outcomes and safety profiles. However, Endo-TLIF shows benefits in reducing blood loss and shortening hospital stays. The shorter operation time of the MIS-TLIF group is also noteworthy. These results offer theoretical guidance for clinicians in choosing surgical methods for treating lumbar degenerative diseases.

### Conclusion

In conclusion, Endo-TLIF and MIS-TLIF are similar in terms of long-term clinical outcomes, fusion rates, and complication rates. While MIS-TLIF has a shorter operation time, Endo-TLIF is advantageous in reducing blood loss, minimizing surgical trauma, hastening recovery, and providing early relief from postoperative back pain. However, due to the limitations in the quantity and quality of the included literature, as well as the sample sizes, there is a need for more high-quality randomized controlled studies. These future studies should not only compare clinical outcomes but also consider other factors such as cost-effectiveness and specific adverse effects to provide a more comprehensive understanding of the comparative merits of these two surgical procedures.

## Data Availability

Follow-up regarding the Effect of different cement distribution in bilateral and unilateral Percutaneous vertebro plasty on the clinical efficacy of vertebral compression fractures is not complete, so the dataset analyzed in this study is not publicly available but is available to the corresponding author on reasonable request.

## Abbreviations

Endo-TLIF: Endoscopic transforaminal lumbar fusion
MIS-TLIF: Minimally invasive transforaminal lumbar fusion
TLIF: Transforaminal lumbar interbody fusion
RCT: Randomized controlled study
ODI: Oswestry disability index
VAS: Visual analog scale
